# Supplementary figures and images for: Prognostic biomarkers related to PANoptosis in esophageal cancer and their immune microenvironment: multi-omics analysis and therapeutic significance
Source: Front Oncol. 2026 Mar 18;16:1755582. doi: 10.3389/fonc.2026.1755582 (PMC13038434; doi:10.3389/fonc.2026.1755582)

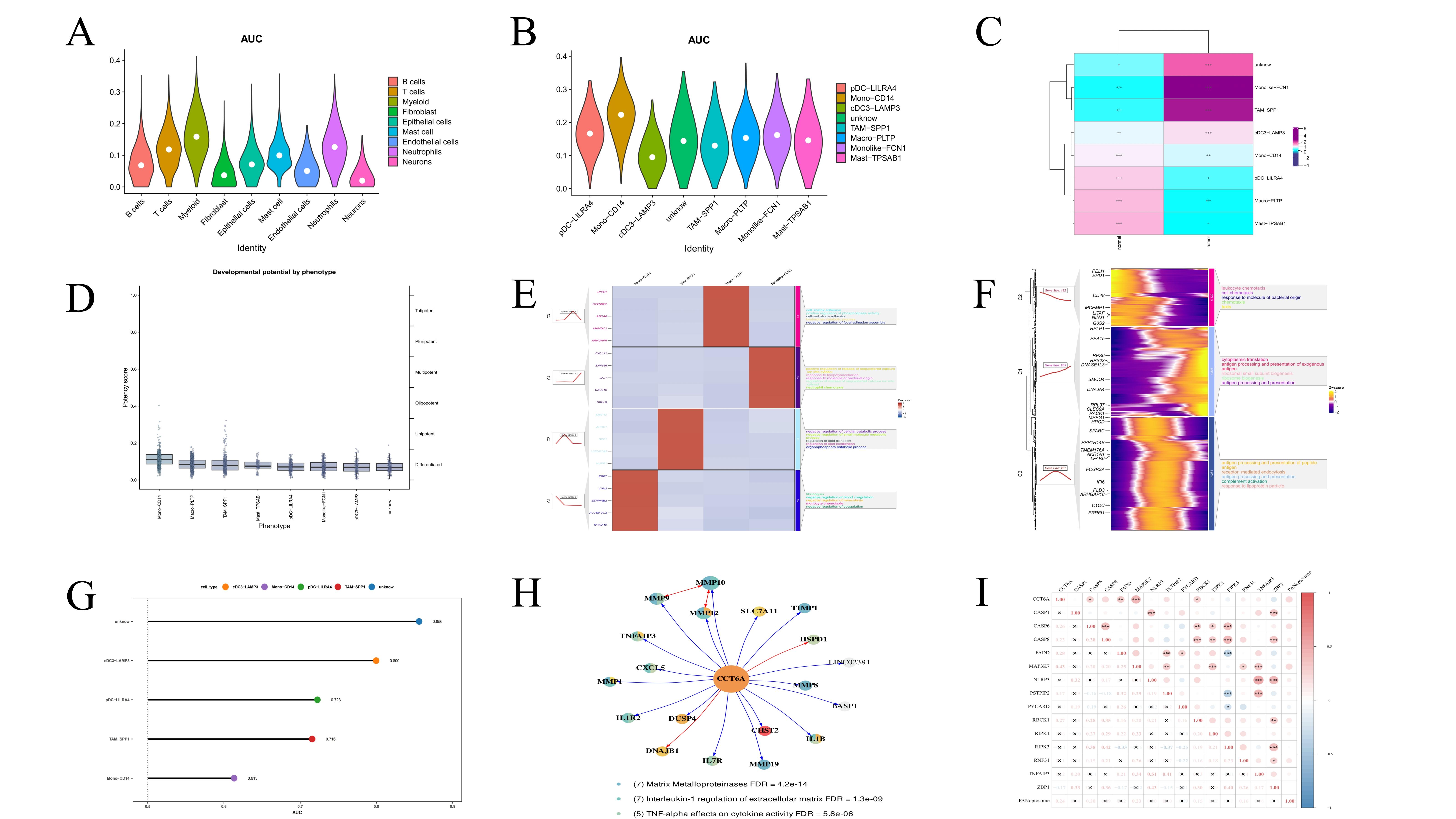

Supplement: Supplementary Figure 1 — Quercetin targets CCT6A to inhibit TAM-SPP1 formation and may be involved in ESCA pan-apoptosis through SPP1/CD44. (A) AUCell score of myeloid cells (B) AUCell score of myeloid cell subsets (C) Ro/e analysis of myeloid cell subsets (D) CytoTRACE stemness score of myeloid cell subsets (E) Mononuclear macrophage lineage top5 markers functional enrichment analysis (F) Dynamic enrichment analysis of cell trajectory gene characteristics (G) Myeloid cell Augur cell perturbation analysis (H) Perturbation gene after virtual knockout of CTT6A in TAM-SPP1 (I) Heat map analysis of correlation between CCT6A, PORGs, and PANoptosome [file Image1.jpeg]
